# Time lag between peak concentrations of plasma and salivary cortisol following a stressful procedure in dairy cattle

**DOI:** 10.1186/s13028-014-0061-3

**Published:** 2014-10-09

**Authors:** Carlos E Hernandez, Tomas Thierfelder, Kerstin Svennersten-Sjaunja, Charlotte Berg, Agustin Orihuela, Lena Lidfors

**Affiliations:** Department of Animal Environment and Health, Swedish University of Agricultural Sciences, P.O. Box 234, SE-532 23, Skara, Sweden; Department of Energy and Technology, Swedish University of Agricultural Sciences, SE-753 23, Uppsala, Sweden; Department of Animal Nutrition and Management, Swedish University of Agricultural Sciences, P.O. Box 7024, , SE-750 07 Uppsala, Sweden; Facultad de Ciencias Agropecuarias de la Universidad Autónoma del Estado de Morelos, Avenida Universidad 1001 Col. Chamilpa, Cuernavaca, Morelos 62210 México

**Keywords:** Calves, Cattle, Cortisol, Cows, Dairy, Plasma, Saliva, Stress

## Abstract

**Background:**

Measurement of salivary cortisol has been used extensively as a non-invasive alternative to blood sampling to assess adrenal activity in ruminants. However, there is evidence suggesting a considerable delay in the transfer of cortisol from plasma into saliva. Previous studies in cattle have used long sampling intervals making it difficult to characterise the relationship between plasma and salivary cortisol (PLCort and SACort, respectively) concentrations at different time points and determine whether or not such a time lag exist in large ruminants. Therefore, the objective of this study was to characterise the relationship between plasma and salivary cortisol and determine if there is a significant time lag between reaching peak cortisol concentrations in plasma and saliva across a 4.25 h time-period, using short sampling intervals of 10–15 min, following social separation in dairy cattle.

Five cows were separated from their calves at 4 days after calving, and six calves were separated from a group of four peers at 8 weeks of age. Following separation, the animals were moved to an unfamiliar surrounding where they could not see their calves or pen mates. The animals were catheterised with indwelling jugular catheters 1 day before sampling. Blood and saliva samples were obtained simultaneously before and after separation.

**Results:**

In response to the stressors, PLCort and SACort increased reaching peak concentrations 10 and 20 min after separation, respectively. This suggested a 10 min time lag between peak cortisol concentrations in plasma and saliva, which was further confirmed with a time-series analysis. Considering the 10 min time lag, SACort was strongly correlated with PLCort (*P* < 0.0001).

**Conclusions:**

Salivary cortisol correlates well with plasma cortisol and is a good indicator of the time-dependent variations in cortisol concentrations in plasma following acute stress. However, there is a time lag to reach peak cortisol concentrations in saliva compared to those in plasma, which should be considered when saliva samples are used as the only measure of hypothalamic-pituitary-adrenal axis response to stress in cattle.

## Background

The assessment of plasma cortisol (PLCort) concentrations has been widely used as an indicator of the activation of the hypothalamic-pituitary-adrenal (HPA) axis in response to stressful situations in cattle [1-4]. However, blood sampling is an invasive method that requires trained personnel and can cause considerable stress to the animals. Because saliva collection is generally less arousing than venipuncture, it has been suggested that salivary cortisol (SACort) concentration is a more appropriate measure of HPA axis activity than PLCort concentrations [5]. Previous studies have shown that salivary and plasma cortisol correlates relatively well in different species including cattle [6], sheep [7], goats [8], pigs [9], dogs [10] and horses [11]. For this reason SACort measurements have been extensively used as a less invasive method to assess HPA axis activity in response to stress in cattle [12,13].

About 80% of the PLCort is bound to corticosteroid-binding globulins (CBG), 10% to albumin and 10% remains unbound (free) [14]. The unbound portion is the biological active fraction of PLCort [15] and the only portion that crosses into saliva. Due to its low molecular weight (362 mol.wt.) and lipophilic nature, unbound cortisol rapidly diffuses through the cell membranes via passive intracellular diffusion [16]. This should allow a relatively rapid equilibrium between salivary and free plasmatic cortisol concentrations [15] which is largely unaffected by salivary flow rate [16]. However, partitioning between the two body fluid compartments is often not simple making correlations between PLCort and SACort sometimes difficult to find. For example, in horses a significant correlation was found only in animals with an oral stereotypy while no significant correlation was found in the control animals [11]. In pigs under different stress conditions, only a weak correlation between PLCort and SACort was found [17] and SACort has been suggested to be a less sensitive indicator of adrenal activity than plasma cortisol in this species [18].

In addition, there is evidence to suggest that there is a time lag between changes in plasma cortisol and associated changes in SACort concentrations of 5.5-7.5 min in humans [19] and 20–30 min in sheep [20]. Although a previous study in cattle [6] did not report a time lag between reaching peak concentrations in plasma and saliva, we hypothesised that their relatively long sampling intervals (20 and 60 min) could have concealed a time lag between changes in PLCort and SACort if the magnitude of the time lag was smaller than their sampling interval. For these reasons, the present study aimed to evaluate the relationship between cortisol concentrations in plasma and saliva using shorter sampling intervals of 10–15 min following a stressful procedure in dairy cattle.

Many routine procedures in commercial dairy farms can be stressful and lead to different physiological and behavioural responses in cattle. For example, handling [21], social separation [22] and isolation in unfamiliar surroundings [23] are stressful situations that can activate the HPA axis leading to an increase in cortisol concentrations in cattle. In Sweden, the legislation for organic farming used to require that the calves stay with the mother during the first 4 days after birth [24] but currently this has been reduced to be at least 24 h in close contact with the mother [25]. The cow and calves are separated after this initial period of close contact; the cows are usually moved from a familiar to an unfamiliar environment and in some farms tethered in stalls during winter. The calves are usually moved to a group of peers. These practices are known to cause strong stress behavioural reactions in the animals [26]. For these reasons, social separation and relocation to unfamiliar surroundings were used to induce an increase in cortisol concentrations in response to stress in cattle.

The objective of this study was to characterise the relationship between plasma and salivary cortisol concentrations across a 4.25 h time-period, and determine if there is a significant time lag between reaching peak cortisol concentrations in plasma and saliva, following social separation in dairy cows and calves.

## Methods

### Animals, management and housing

All procedures were approved by the Ethical Committee of Experimental Animals of Gothenburg, Sweden. The study was carried out on a commercial organic dairy farm, in the southwest of Sweden. The farm had approximately 300 Swedish Holstein (SH) and Swedish Red (SR) dairy cows in a cubicle-based loose housing system with a parallel milking parlour where cows were milked twice a day. In an adjacent building, dry and sick cows were tethered in long stalls. On the other side of the manger, a group of foster cows were kept in a communal pen, tethered in long stalls and kept with a group of calves that ran free within a fenced area along the stalls. The concrete floor of the stalls was covered with rubber mats and bedded with wood shavings. The cows were fed a Total Mixed Ration twice per day and had *ad libitum* access to water in water bowls.

The present study was conducted during summer (June to August) so the cows were kept on pasture with their calves for 2 days after calving. On the third day after calving, cow-calf pairs were housed in individual indoor pens until 4 days of age. The individual indoor pens (3 × 3 m) had a concrete floor with rubber mats and bedding of wood shavings. The cows had free access to water from a nose-press water bowl and mineral salts. At 4 days of age, the calves were moved to the communal pen with a group of foster cows and were kept there until 7 weeks of age. From 7 to 12 weeks of age, the calves were moved into groups of four calves and kept in a group pen where half of the concrete slatted floor was covered with rubber mats and wood shavings for comfort. During this period, the calves were bucket fed 3 l of whole milk in the morning (0800, except on the day of sampling when calves were feed at 0700) and 3 l in the afternoon (1400). The animals had free access to water from a nose-press water bowl and mineral salts; the feed consisted of a Total Mixed Ration plus concentrate.

### Treatments

Five cows at 4 days postpartum and six calves at 8 weeks of age (±2 days) were used in the experiment. The cows and calves used in the experiments were not related to each other. Four cows were in their first lactation (two SH and three SR) and one was in her second lactation (SH). One calf was male (SH) and five were females (three SH and two SR).

Jugular catheters were placed on the cows (but not their calves) at 3 days postpartum and on the 8 week-old calves one day before sampling.

The procedures for the 4.25 h blood and saliva sample collection were as follows:

A) On the 4th day after calving, cows were separated from their calves and tethered in the building used for foster cows and calves (unfamiliar surrounding) and located 20–25 m from their indoor home pen. The cows were tied up leaving two stalls empty between the two cows to be sampled and several stalls away from other cows. The cows remained tethered until the end of the sampling. The animals had free access to water from a nose-press water bowl and feed was available as a Total Mixed Ration. Their 4-day-old calves remained in their indoor home pen and were not used for any samples.
B) At 8 weeks of age, calves were separated from their group and moved to unfamiliar individual pens (1 × 1.25 m) located 8–10 m away from their home pen. Calves were kept there until the end of the sampling. In this pen, the calves could have auditory and visual, but no physical, contact with the neighbouring calf. The calves had free access to water and concentrate from two metal bowls hanging on the outside of the pen and hay was available from racks hanging on the wall of the pen. Each individual pen was supported by four metal legs 55 cm above the floor and had plastic slatted floors with straw bedding.

### Sampling design

Plasma and saliva samples were collected from no more than two animals on the same day. Samples were taken by two persons in order to obtain blood and saliva at the same time. Blood and saliva samples were taken for basal levels starting at 08:00 from cows (in the calving pen) and calves (in the group pen). Another sample was taken 10 min later, and the animals were immediately separated and moved to an unfamiliar surrounding. Moving and tethering the cows in the unfamiliar surrounding took less than 5 min. Next samples were taken 15 min after the previous sample. Further samples were taken at 10 min intervals for the next 110 min and every 15 min for the last 105 min of sampling. During the 255 min sampling sequence, 22 blood and 22 saliva samples were taken simultaneously at −10, 0, 15, 25, 35, 45, 55, 65, 75, 85, 95, 105, 115, 125, 140, 155, 170, 185, 200, 215, 230 and 245 min after separation.

### Sampling procedures

#### Jugular catheters

The animals were tranquilised with Xylazin hydrochloride (i.v. 0.05 mg/kg; Narcoxyl Vet; 20 mg/ml, Intervet international B.V., P.O. Box 31, 5830 AA Boxmeer N.) for jugular catheterisation. The catheter (length 105 mm, i.d. 1.5 mm, o.d. 2.0 mm; Intranule, Laboratoires pharmaceutiques, France) was inserted toward the heart into the jugular vein, sutured to the skin and fitted with 140 cm of Heidelberg extension tubing (B. Braun Melsungen AG, D-34209, Melsungen). The free end of the extension tube was sutured to the back of the animals and a three-way stopcock (SARSTED, Aktiengesellschaft & Co., D-51588 Nümbrecht, Germany) was fitted to facilitate the frequent sampling. The system was filled with heparinised saline solution (50 IU/ml dilution, LEO Pharma AB, 201 24 Malmö, Sweden), taped to the neck and covered with a self-adhesive elastic band.

#### Blood samples

Two volumes of the sampling catheter and extension tube were discarded before the withdrawal of a 10 ml blood sample for cows and 5 ml blood sample for calves. This part of the procedure took approximately 30 s. Immediately after this, the catheter was flushed (two volumes of the catheter and extension tube) with saline solution (0.9% NaCl). In addition, the catheter was flushed with heparinised saline solution every hour. Blood was collected with sterile syringes and immediately decanted into evacuated blood collection tubes containing sodium heparin (Venoject, Terumo Europe N.V., 3001 Leuven, Belgium). The samples were placed in an ice bath until centrifugation at 3000 × g for 15 min (within 2 h after collection) and the plasma immediately frozen at −20°C.

#### Saliva samples

Saliva samples were collected at the same moment as the blood samples were taken. A cotton swab (Salivette; SARSTEDT, Aktiengesellschaft & Co.) was held with metal forceps and placed inside the mouth of the cows and calves until saturated (approx. 1 ml, 1 to 2 min). The saliva samples were stored on ice, centrifuged at 4500 × g for 15 min (within 2 h after collection) and immediately frozen at −20°C until analysis for cortisol.

#### Assay

PLCort and SACort concentrations were determined using a solid phase ^125^I-RIA (Coat-A-Count RIA kit, Diagnostic Products Corporation, Los Angeles, CA, USA) following standard procedures. The intra-assay CV was below 10% for both, plasma and saliva samples. The inter-assay CV ranged from 7.2 to 8.7% for plasma and from 4.0 to 6.6% for saliva. The detection limit of the assay was 0.252 nmol/l for plasma and 2.8 nmol/l for saliva. The assay has previously been validated for bovine cortisol determination with a recovery of 91% in plasma and 84.5% in saliva by the laboratory at the Department of Anatomy and Physiology at the Swedish University of Agricultural Sciences (SLU, Uppsala).

### Statistical analysis

Data were analysed using the Variance Estimation and Precision (VEPAC) toolbox of STATISTICA software version 8.0 (StatSoft Inc., Tulsa, USA), whereas the Curve Fitting toolbox of MATLAB version 7.1 (MathWorks Inc. Natick, USA) was used for the interpolation of time-series. Data are presented as means ± S.E.

Pre-whitened time series (see results section “Time lag between plasma and salivary cortisol”) of PLCort and SACort were used in order to establish a mixed general linear model wherein the relationship given in equation 1 (see results section “Inter-distance between observations”) was tested for validity across factors of age, breed, and individual identity. The model included PLCort as response variable, SACort as a covariate (shifted one time-step ahead), age (cow or calf) and breed (SH or SR) as fixed factors, and animal ID as a random factor. In a restricted maximum likelihood approach, the estimation of fixed-factor effects was conditioned with the within-subject covariance assumed to prevail within animal individuals (i.e. stronger covariance within than across subjects).

## Results

### Age and breed differences in cortisol

PLCort and SACort concentrations were similar in cows and calves and in SR and SH animals. Individual variation in PLCort and SACort response to the stressors accounted for 37% of the total variance observed (this relatively large effect was compensated throughout all tests performed). Therefore, data was analysed together considered as homogenous with respect to age and breed, and the statistical analysis was focused on the distribution across time-steps and on individual animals.

### Time lag between plasma and salivary cortisol

**Figure 1:**
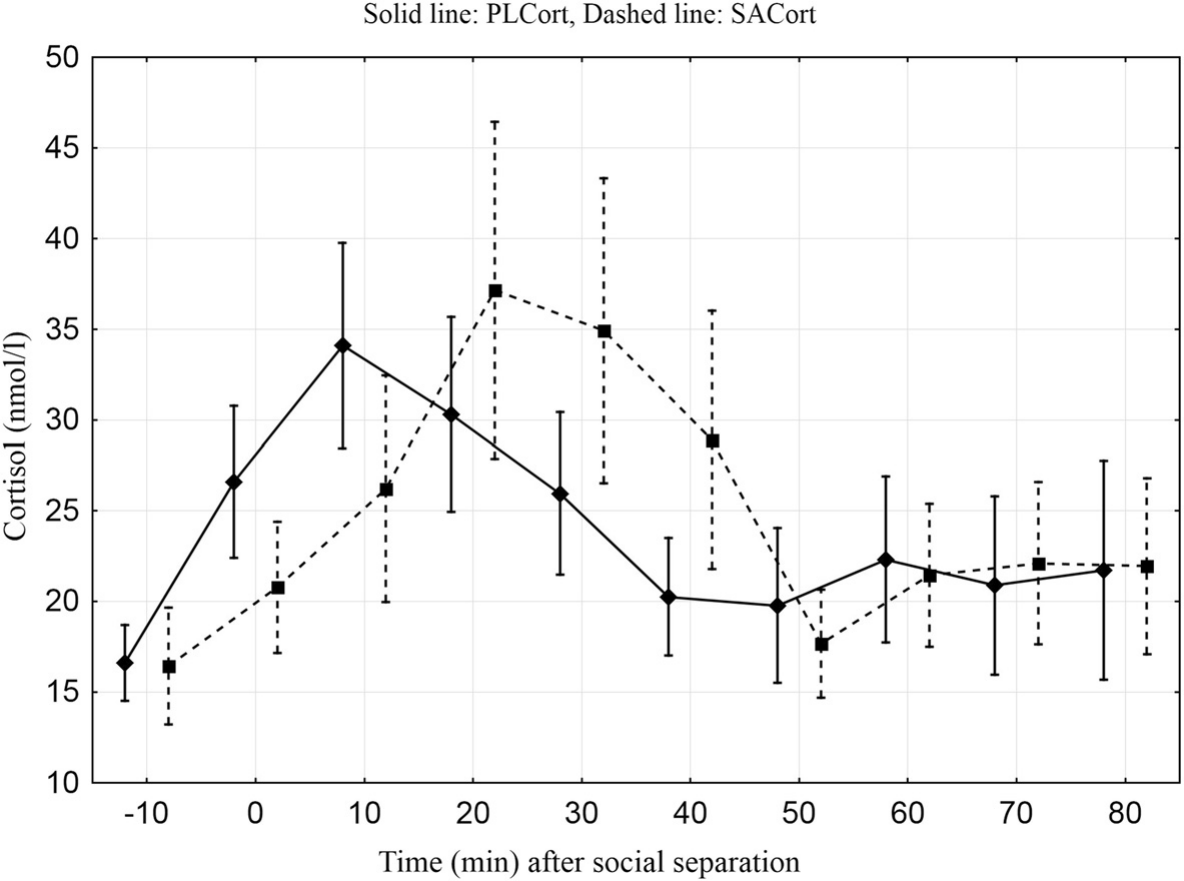
**Plasma (PLCort) and salivary (SACort) cortisol concentrations (nmol/l) through a 90 min sequence with time zero allocated to the time of social separation.** Values averaged across cow and calf subjects, given with standard errors. Time-series interpolated to 10 min equidistance with SACort concentrations multiplied by a factor of 10 for clarity. The series are slightly time-shifted to avoid overlapping error bars.

**Figure 2:**
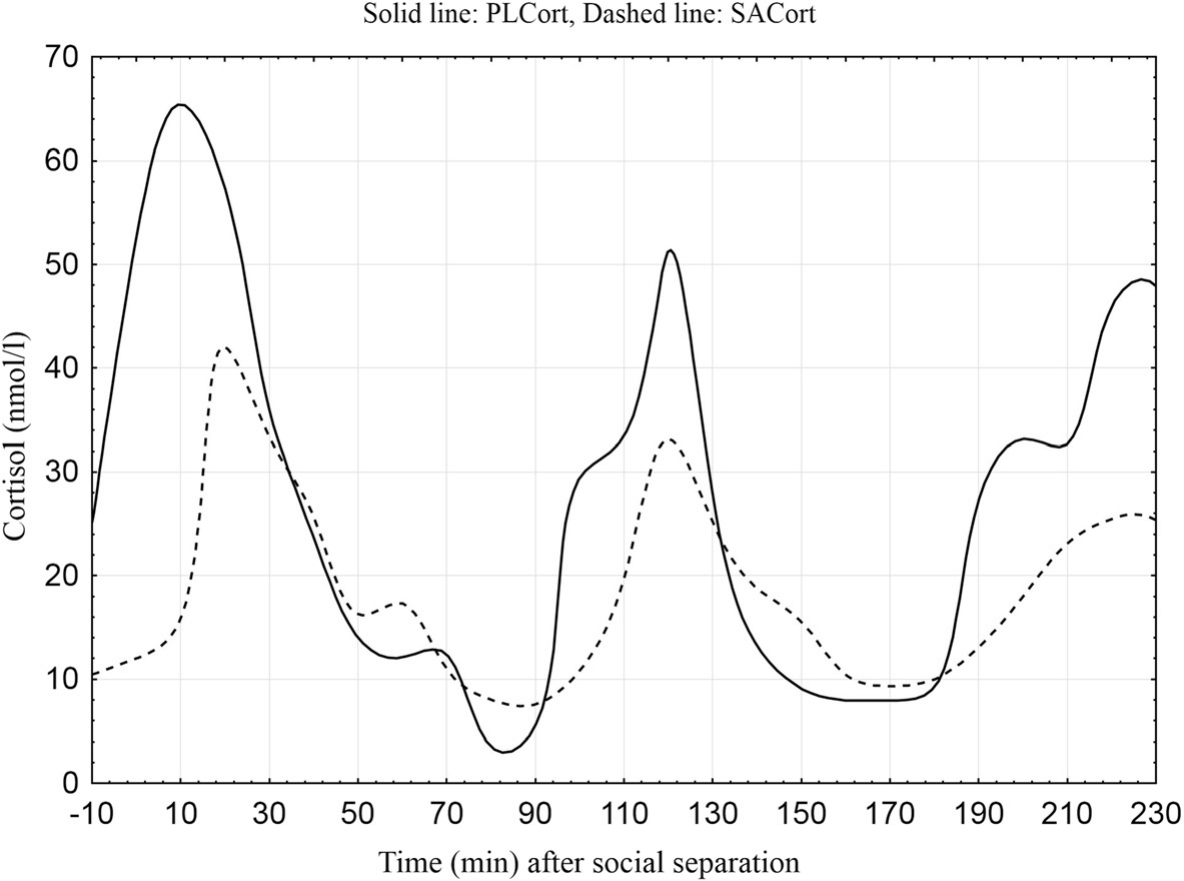
**Plasma (PLCort) and salivary (SACort) cortisol concentrations (nmol/l) of calf id. 1592 throughout the experiment, with stressor introduced at time zero.** Individual time-series values interpolated to 10 min equidistance with SACort concentrations multiplied by a factor of 10 for clarity.

Peak mean values of PLCort (34.11 ± 5.66 nmol/l) and SACort (3.49 ± 2.79 nmol/l) were reached 10 and 20 min after social separation, respectively (Figure 1). The increase in SACort was lagged compared to the rise PLCort but the decline in cortisol concentrations occurred approximately at the same time in both fluids. To illustrate this PLCort and SACort values from one calf are shown (Figure 2).

**Figure 3:**
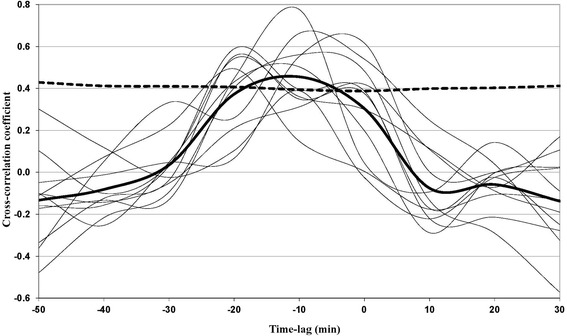
**Cross-correlation coefficients of all subjects (thin lines) together with the average cross-correlative curve (bold line) and the upper 95% confidence limit (dashed line).** When averaged across all subjects, the correlation across plasma (PLCort) and salivary (SACort) cortisol peaks at a time-shift of approximately –12 min, with PLCort preceding SACort. The average correlation is non-significant (at the 95% level) with zero time-shift.

To test the hypothesis that cortisol concentrations in saliva were negatively time-lagged compared with plasma cortisol concentrations, a successively time-lagged spectra of correlations across plasma and saliva cortisol sequences was established (Figure 3). The standard method for accomplishing this is using cross-correlative autoregressive integrative moving average (ARIMA) models [27], which require that the cortisol sampling sequences are obtained on an equidistant basis. Since they were not, a shape-preserving interpolant piecewise cubic Hermite interpolation (PCHIP) curve was fitted to the data [28], to allow parsimonious interpolation of equidistant time-series equivalents. As a result, 10 min equidistant curves ranging from −10 to 240 min were calculated with 10 interpolated plus 16 primary values per curve. With time-series being characterised by their internal correlation structure (auto-correlation), it is impossible to know whether the observed saliva response depends on plasma cortisol or on the previous cortisol-state within the observed saliva time-series. In order to isolate the plasma effect, internal correlation structures must be filtered out (pre-whitening) prior to the establishment of a cross-correlative spectra [29].

With all response curves accordingly pre-whitened, the individual cross-correlative spectres showed that the cortisol response of dairy cattle saliva was significantly lagged as compared with the cortisol concentrations in plasma (Figure 3). The PLCort concentrations preceded the corresponding concentrations of SACort with 0–20 min with an average peak of significance at approximately −12 min. A good correlation was still apparent when allowing a 20 min time lag between PLCort and SACort but vanished thereafter (Figure 3). Allowing a 10 min time lag, PLCort strongly correlated with SACort (R^2^ = 0.83, F_(1, 22)_ = 113, *P* < 0.0001).

### Inter-distance between observations

Since the strength of autocorrelation depends on the inter-distance between observations, and since pre-whitened time series are composed by approximately non-correlated (independent) observational equivalents to the original cortisol sequences [30], pre-whitened time series are robust with respect to observational inter-distance. Therefore, irrespective of sampling frequency, they can be used to establish a predictive model where the PLCort concentrations may be estimated on basis of the corresponding SACort concentrations. When measured at the scale of minutes, equation 1 holds:

1 $$ {\mathrm{PLCort}}_{\mathrm{t}}=4.47 + 8.09 \times {\mathrm{SACort}}_{t-10}\ {{\mathrm{R}}^2}_a={0.83}_{,}{\mathrm{F}}_{\left(1,\ 22\right)}=113 $$

Since equation 1 establishes a relationship across two parallel time-series shifted 10 min apart, the regression intercept expresses some transient baseline relation across two distinct temporal positions. When, in the more general case, average concentrations are compared across time-series, the regression intercept should be forced to zero. As a result, the SACort concentration amounts to 9.86 ± 0.64% of the PLCort concentration.

## Discussion

In this study, we characterised the relationship between plasma and salivary cortisol concentrations and tested the hypothesis that there is a time lag between peak concentrations of plasma and salivary cortisol after a stressful procedure in dairy cattle. We found that there is a time lag of approximately 10 min to reach peak cortisol concentrations in saliva compared to plasma. This suggests a delay to reach equilibrium between cortisol concentrations in both fluids after a stressful event in dairy cattle. Our results are similar to the ones reported in humans [19] and sheep [20] where a time lag of 5.5–7.5 min and 20–30 min, between cortisol concentrations in plasma and saliva were found, respectively. In dogs, there is also evidence of a delay in the increase of cortisol concentrations in saliva compared to those in plasma; however the time lag could not be quantified due to long sampling intervals [31]. These studies and the results reported in our study suggest that the time lag between PLCort and SACort concentrations may vary between species. However, these differences could also reflect differences in sampling intervals and sampling procedures between studies.

In a previous study in cattle, there was a good correlation between PLCort and SACort, however no time lag was reported [6]. The absence of a time lag between cortisol values in both fluids could be due to the blood and saliva sampling scheme, the procedures used in their study and the fact that PLCort and SACort concentrations correlate well at different time points along the response curve. In the study by Negrao *et al*. [6], the samples were taken every 20 min during the first hour and every hour for another 5 h, and the saliva samples were taken only after each blood sample had been obtained. In our study, blood and saliva samples were taken simultaneously and the samples were taken every 10 min for the first 2 h. This relatively short sampling interval along with simultaneous blood and saliva sampling allowed us to obtain a better image of the variation in cortisol concentrations between both fluids and of their relationship across time. The time lag found in our study should be considered in future studies assessing cortisol concentrations in saliva in response to stress in dairy cattle by allowing a 10 min time lag from the expected time to reach peak cortisol concentrations in plasma or by using regular short sampling intervals of 10 min.

The mechanism underling the time lag between reaching peaks in PLCort and SACort observed in the present study is unknown. However, there are some factors known to affect the disposition of free cortisol in plasma. For example, plasma corticosteroid-binding globulins (CBG) [14,32-34], 11β-hydroxisteroid dehydrogenase in saliva and salivary glands [35,36], temperature and pH [37] all can affect the disposition of free cortisol. In humans, the binding of cortisol to CBG has been suggested as a likely mechanism to explain the delay in reaching peak levels in salivary cortisol compared to plasma cortisol [19]. Because the activity of CBG is similar in humans and other animal species including dairy cows [38], it is likely that CBG effects on free cortisol could explain our results. It is clear that several conditions can affect the disposition of free cortisol and this may explain the difficulties to find good correlations between cortisol values in plasma and saliva reported in different species.

In response to social separation and restraint in unfamiliar surroundings, the cows and calves showed an apparent increase from the basal concentrations in PLCort and SACort. Similar results have been reported in cattle where cortisol concentrations increased in response to social separation [22], unfamiliar surroundings [39] and social isolation in unfamiliar surroundings [23]. However, in the present study it is unclear which factors determined the subsequent peaks in cortisol concentrations and the variations observed over time. The HPA axis can respond to a wide range of psychological and physiological stimuli [40] making it difficult to determine the cause of the subsequent cortisol peaks and variations over time. The animals in this experiment were not used to handling and restraint and none of the cows in the present study had been tethered before. Handling [41] and tethering [42] are known to be stressful to the cows and lead to increases in plasma cortisol concentrations and may explain the subsequent peaks in cortisol concentrations observed after the initial stressors. Cow-calf separation within the first week after calving is known to affect the behaviour of the cows [43] but not the plasma cortisol concentrations [4]. Therefore, cow-calf separation is unlikely to be a major cause for the cortisol increase observed in our study. Marked ultradian rhythms (of about 120 min) of cortisol concentration have been reported for cows [44]. However, only the more sustained increases are associated with situations of stress [45], and in this study, samples were taken at the same time of the day to avoid any possible confounding effects of circadian and ultradian rhythms of the cortisol secretion so it is unlikely that circadian patterns affected our results.

## Conclusions

Social separation and unfamiliar surroundings are stressful situations that result in an increase in cortisol secretion in cattle. This response can be evaluated through salivary cortisol, which in addition to being less invasive than blood sampling, seems to be a good estimate of plasma cortisol following activation of the HPA axis in response to acute stress in dairy cattle. However, there is a time lag to reach peak cortisol concentrations in saliva compared to those in plasma that should be considered when using saliva samples as the only measure of stress responses in cattle.

## Abbreviations

PLCort: Plasma cortisol
SACort: Salivary cortisol
CBG: Corticosteroid-binding globulins
SH: Swedish Holstein
SR: Swedish red
HPA: Hypothalamic-pituitary-adrenal
